# Outer membrane vesicles in gram-negative bacteria and its correlation with pathogenesis

**DOI:** 10.3389/fimmu.2025.1541636

**Published:** 2025-04-01

**Authors:** Fatemeh Sadat Abolhasani, Nasim Vaghefinanekaran, Aref Yarahmadi, Sousan Akrami, Solmaz Mirmahdavi, Mohammad Hasan Yousefi, Hamed Afkhami, Morvarid Shafiei

**Affiliations:** ^1^ Department of Pathobiology, School of Public Health, Tehran University of Medical Sciences, Tehran, Iran; ^2^ Department of Microbiology, North Tehran Branch, Islamic Azad University, Tehran, Iran; ^3^ Department of Biology, Khorramabad Branch, Islamic Azad University, Khorramabad, Iran; ^4^ Department of Microbiology, School of Medicine, Tehran University of Medical Sciences, Tehran, Iran; ^5^ Department of Bacteriology and Virology, Faculty of Medicine, Tabriz University of Medical Sciences, Tabriz, Iran; ^6^ Department of Microbiology, Faculty of Medicine, Shahrekord University of Medical Sciences, Shahrekord, Iran; ^7^ Student Research Committee, Qom University of Medical Sciences, Qom, Iran; ^8^ Department of Tissue Engineering and Applied Cell Sciences, School of Medicine, Qom University of Medical Sciences, Qom, Iran; ^9^ Cellular and Molecular Research Center, Qom University of Medical Sciences, Qom, Iran; ^10^ Nervous System Stem Cells Research Center, Semnan University of Medical Sciences, Semnan, Iran; ^11^ Department of Medical Microbiology, School of Medicine, Shahed University, Tehran, Iran; ^12^ Department of Bacteriology, Pasteur Institute of Iran, Tehran, Iran

**Keywords:** outer membrane vesicles (OMVs), gram-negative bacteria, pathogenesis, immune system, vaccines, secretion system

## Abstract

There is a widespread distribution of gram-negative bacteria worldwide, which are responsible for the deaths of numerous patients each year. The illnesses they cause can be localized and systemic, and these bacteria possess several key virulence factors that contribute to their pathogenicity. In recent years, several distinct mechanisms of pathogenesis have evolved that remain largely unknown to scientists and medical experts. Among these, outer membrane vesicles (OMVs) are undoubtedly one of the most significant factors influencing virulence. OMVs contain various bacterial compounds and can have diverse effects on host organisms and the immune system, potentially exacerbating disease and inflammation while evading immune responses. This review comprehensively examines the role of OMVs in bacterial pathogenesis, their interaction with host cells, and their potential biomedical applications. Understanding the molecular mechanisms governing OMV biogenesis and function could pave the way for novel antimicrobial strategies and therapeutic interventions.

## Introduction

The outer membrane vesicles (OMVs) released by outer membrane bacteria are two-layer small spherical vesicles (1). At first, they were reported more than 50 years ago in studies on bacterial growth (2). OMVs carry lipopolysaccharide (LPS), phospholipids, peptidoglycan (PG), cell wall components, outer membrane proteins (OMP), ionic metabolites, periplasmic, cytoplasmic and membrane proteins, signaling molecules, and nucleic acids (DNA and RNA) (3–5).

**Figure 1:**
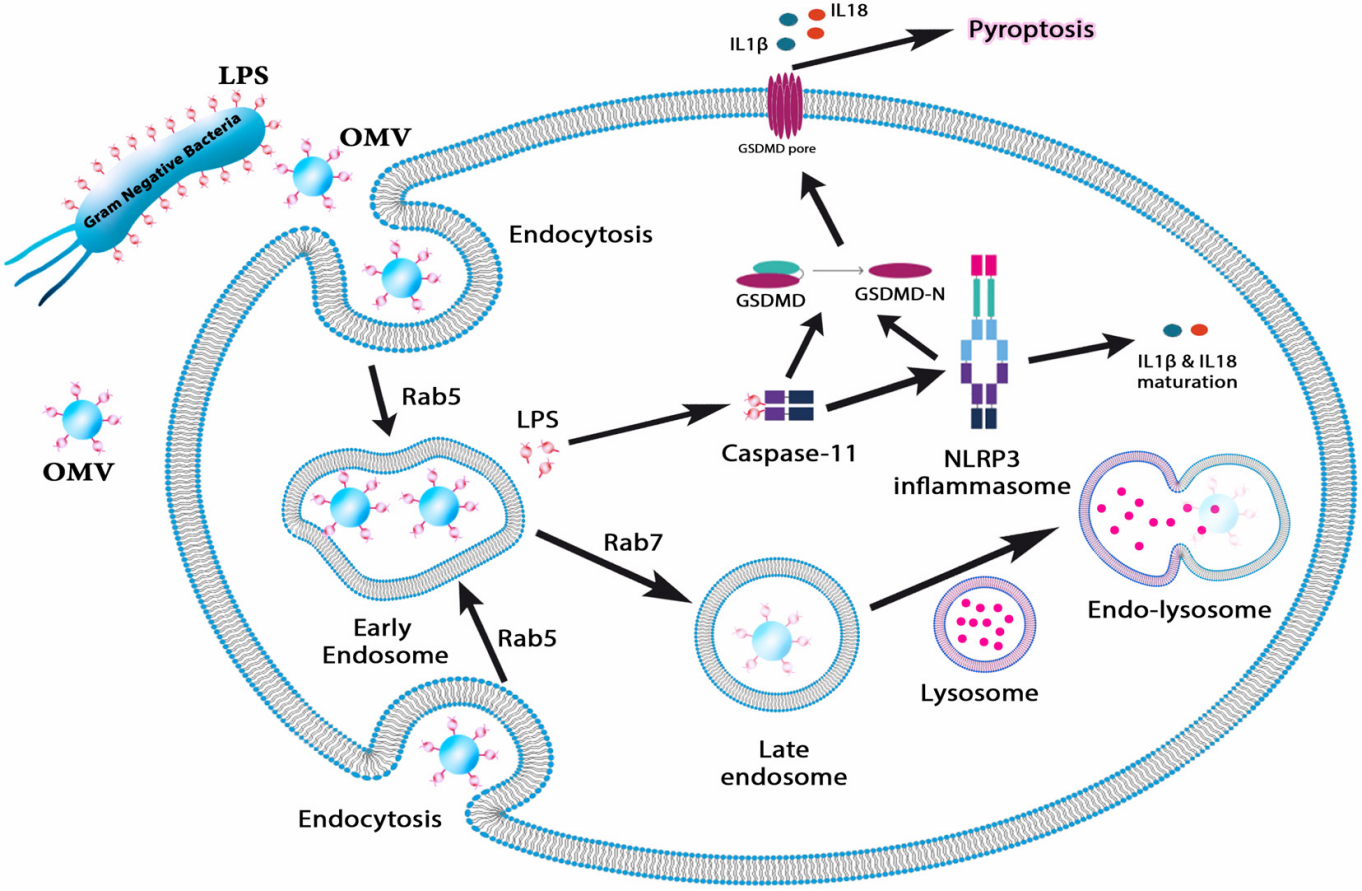
This figure illustrates the role of OMVs in immune activation and pyroptosis. OMVs carrying lipopolysaccharide (LPS) are internalized by host cells via endocytosis, where Rab5 and Rab7 regulate their trafficking through early and late endosomes. LPS escapes from endosomal compartments into the cytosol, activating caspase-11 and the NLRP3 inflammasome. This triggers the maturation of proinflammatory cytokines IL-1β and IL-18, ultimately inducing pyroptosis—a form of programmed inflammatory cell death. This pathway underscores the role of OMVs in bacterial pathogenesis and immune response modulation.

OMVs are generated by both pathogenic and non-pathogenic bacteria (6) and fundamentally influence bacteria-host interactions (7). The functions of OMVs include resistance to antibiotics, adherence to the host, formation of biofilms, delivery of molecules and virulence factors, and modulation of immune system responses (7, 8). OMVs facilitate gram-negative bacteria’s entry into the bloodstream (8). By transferring virulence factors, bacteria can survive in the host environment longer. Some reported virulence factors help the bacteria adapt physiologically and metabolically in hostile environments. In contrast, others are released and carry out a variety of biological and immunological modulations within the host (9). Additionally, these vehicles carry toxins, immunomodulatory compounds, and adhesion molecules (8). According to **Figure 1**, one of the OMVs’ functions is observed (5).

Bacterial OMVs promote intracellular LPS localization, active caspase-11-mediated cytosolic LPS sensing, and endocytosis. Thus, OMVs are required for caspase-11 activation during bacterial infections (5). Environmental forces aid in the production of OMVs (10). When bacteria are exposed to stresses, the synthesis of OMVs may not only alter interactions with the host but also aid in the bacterium’s survival. While a significant aspect of OMV research is aimed at understanding the host recognition of antigenic OMV-bound factors, particularly for vaccine development, the native activity of proteins on OMVs may also play distinct roles in pathogenesis and the modulation of host defense during bacterial infection (6). Other applications of OMVs include drug delivery mechanisms or cancer immunotherapy. In 1997, Bermudes and his colleagues found that *Salmonella* can be a novel drug delivery platform for targeting cancer (11). However, the toxicity of bacteria limits their clinical translation as anticancer carriers in cancer immunotherapy. OMVs have a mixture similar to bacteria. Additionally, manipulated engineered attenuated bacteria can produce OMVs with reduced endotoxicity. Therefore, weakened OMVs have practical value in cancer immunotherapy (12–14).

**Figure 2:**
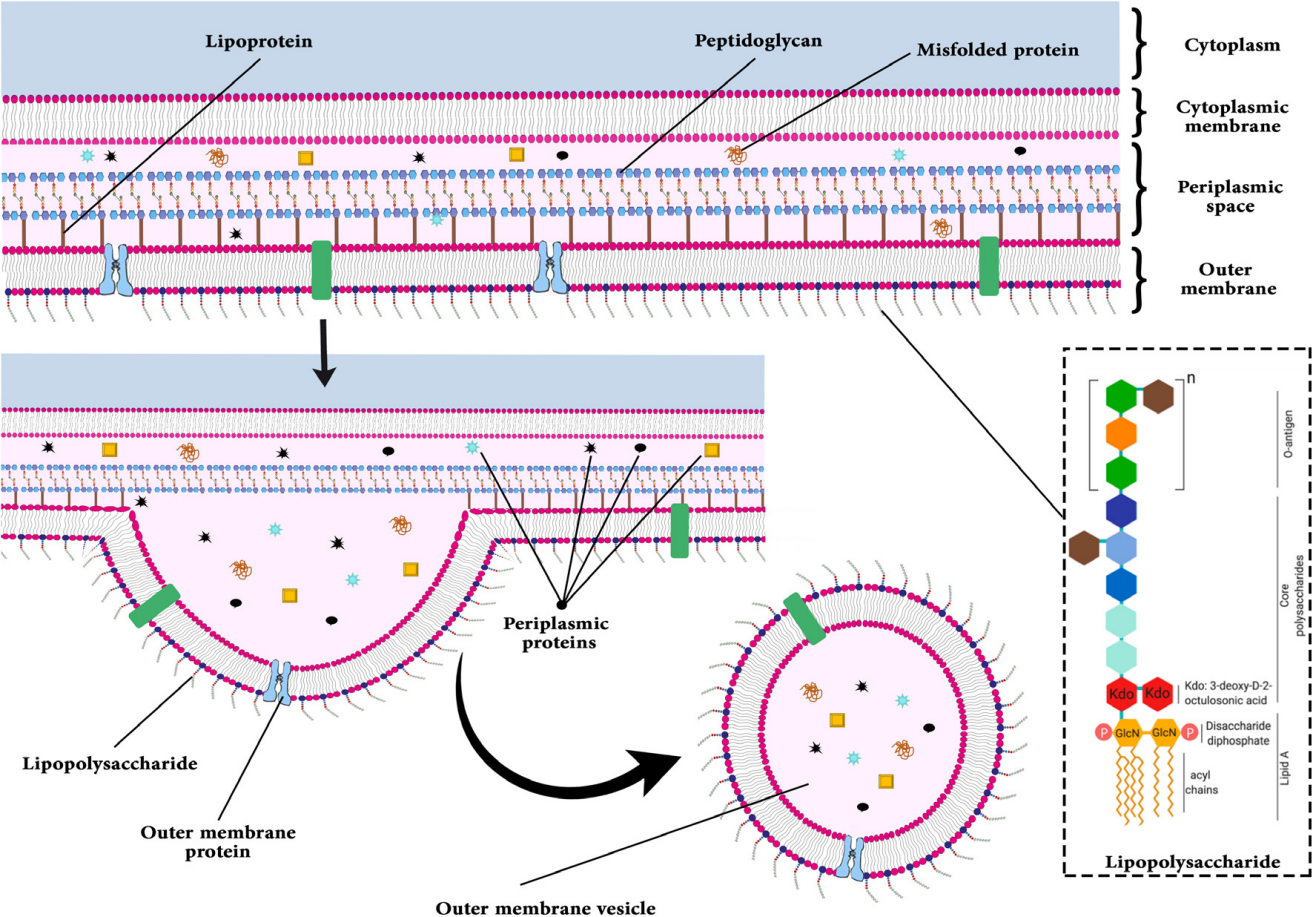
Model of OM vesicle production.

Several bacteria, including *Pseudomonas aeruginosa, Escherichia coli, Shigella* spp.*, Salmonella* spp.*, Vibrio* spp.*, Helicobacter pylori, Campylobacter jejuni, Borrelia burgdorferi*, and *Neisseria* spp. can produce OMVs (7, 15). Several proteins in OMVs lead to increased attachment, internalization, and bacterial invasion. Localized OMV invasions are best exemplified by *E.coli* Ail protein and *Shigella flexeneri* IpaB, IpaC, and IpaD (16). In OMVs, OspA and OspB facilitate the attachment of bacteria to the host’s receptors (17). Several toxins are secreted by bacteria after this step, including Shiga toxin (STx1 and STx2), heat-labile toxin (LT), cholera toxin (CT), vacuolating toxin (VacA), and virulence factors, including glycoproteins and proteases, thereby evading immune responses and modulating host immunity (8). **Figure 2** illustrates the budding process of the gram-negative bacterial envelope. Released OMVs contain periplasmic material, outer membrane proteins and lipids, including pathogen-associated molecular patterns (PAMPs) and other virulence factors. Although the exact mechanism remains unclear, it is believed that budding occurs at sites where the lipoprotein connections between the outer membrane and the PG layer are either damaged or absent (18).

## Constituents of OMVs

At first, OMVs were simply thought to be growth artifacts or cell lysis byproducts (19). The discovery of OMVs in cerebrospinal fluid samples from meningococcal infection patients marked a paradigm shift. It raised the possibility that OMVs are not only created in lab settings but potentially contribute to the progression of illness (20). Researchers are now interested in examining the biogenesis and function of different bacterial OMVs due to this discovery. Terry J. Beveridge and his team conducted groundbreaking research that demonstrated that OMVs gave parental bacteria selection benefits by encouraging the creation of biofilms and acting as transporters of DNA and virulence proteins, which are crucial for genetic change and illness (21, 22).

OMVs are spherical nanoparticles characterized by a lipid bilayer structure, typically ranging in diameter from 20 to 400 nm. These vesicles encapsulate components from the periplasmic space and are formed through a budding process during the active growth phase of bacteria rather than resulting from bacterial lysis (23). OMVs are produced when a segment of the outer membrane of a bacterial cell wall expands, subsequently constricting and releasing. Proteomic and biochemical investigations have demonstrated that OMVs encompass a variety of bacterial constituents, including DNA, RNA, LPS, proteins, enzymes, and PG. Consequently, OMVs are composed of numerous biological materials that are characteristic of the parent bacteria, albeit in a non-replicative state (24, 25).

## Nucleic acid

Renelli et al. (22) discovered that OMVs contain not only luminal DNA but also DNA associated with their surfaces. Following the digestion of exogenous DNA with DNase, the luminal content was found to consist solely of plasmid DNA, which exhibited notable resistance. This observation implies that the DNA in OMVs possesses a degree of resilience that contributes to its stability in external environments. Additionally, various forms of luminal DNA have been identified across a range of bacterial species, including *Pseudomonas aeruginosa*, *Haemophilus influenzae*, and *Neisseria gonorrhoeae* (26–28). Other research has discovered that OMVs include non-coding RNA, mRNA, and microRNA in addition to DNA. OMVs derived from uropathogenic strains of *Escherichia coli* (UPEC) encompass a variety of RNA species, including ribosomal RNA (rRNA), transfer RNA (tRNA), and messenger RNA (mRNA), as reported by Blenkiron et al. Recent studies indicate that *Pseudomonas aeruginosa* utilizes the small RNA (sRNA) present in its OMVs to modulate the host immune response (29, 30).

According to this research, OMVs can act as both transmitters and carriers of genetic information. However, the biological consequences of DNA and RNA transferred to host cells via OMVs are poorly understood. In the context of *Pseudomonas aeruginosa* OMVs, sRNAs have been shown to interact with host mRNA, thereby inhibiting the secretion of cytokines such as IL-8 and reducing neutrophil infiltration, which ultimately diminishes host immune responses (31). Furthermore, the three most prevalent sRNAs identified within these OMVs—sRNA4518698, sRNA2316613, and sRNA809738—are non-coding RNA fragments that exhibit resistance to degradation by external ribonucleases (32).

## Phospholipid

The lipids present in OMVs predominantly consist of phospholipids and LPS, with the phospholipids exhibiting a composition akin to that of the outer membrane (33). Research conducted by Hoekstra et al. (34) demonstrated that the phospholipid profile of Escherichia coli OMVs closely resembles that of the outer membrane. Furthermore, Kato et al. (35) utilized thin-layer chromatography to identify LPS, phosphatidylethanolamine, and cardiolipin as the principal lipid constituents of these vesicles, paralleling the primary lipid components found in the outer membrane. Nevertheless, a comprehensive quantitative lipidomic analysis of the various classes of phospholipids has yet to be undertaken, representing a prospective avenue for future research.

Furthermore, the curvature of the OMVs is significantly greater than that of their bacterial progenitors, suggesting a distinct composition of various phospholipids (36). Tashiro et al. (37) observed a high abundance of phosphatidylethanolamine in the outer membrane, whereas this phospholipid was also present in OMVs. Additionally, the relative proportion of saturated fatty acyl chains is elevated in OMVs compared to the outer membrane, contributing to the increased rigidity of OMVs relative to the outer membrane (37).

## LPS

LPS constitutes a significant element of the outer membrane in gram-negative bacteria and is crucial for the formation and functionality of OMVs. Research indicates that not all LPS molecules produced by bacteria are incorporated into OMVs; instead, only specific types of LPS are found within these vesicles. Consequently, LPS may have a vital role in the biogenesis of OMVs (38). Kadurugamuwa et al. (39) have indicated that the OMVs of *Pseudomonas aeruginosa* primarily comprise the negatively charged B-band LPS, as opposed to the more neutral A-band LPS. The findings of this research imply that LPS may be significant in the biogenesis of OMVs. Furthermore, LPS is not only found within OMVs but also serves a crucial function in their formation. It is known that some chemical forms of LPS, including pentanoylated LPS produced by PagL, cause bacteria to release more OMVs, both in terms of quantity and size. According to this, altering LPS may promote the synthesis of OMVs. Furthermore, the B-band form of charged polysaccharides is the primary form of LPS frequently seen in OMVs, and its enrichment in OMVs is essential for the production of OMVs (40).

Given the diverse molecular composition of OMVs, their role extends beyond structural components to actively influencing bacterial pathogenesis. The ability of OMVs to mediate horizontal gene transfer, modulate host immune responses, and contribute to antimicrobial resistance underscores their significance in bacterial survival and virulence (18, 41). The following sections explore the role of OMVs in key bacterial pathogens, highlighting their contributions to disease progression and host interactions.

## Role of OMVs in bacterial pathogenesis

### Acinetobacter baumannii

In humans, *Acinetobacter baumannii* (*A. baumannii*) is a pleomorphic aerobic gram-negative bacillus that is non-motile, non-fastidious, catalase-positive, and an opportunistic pathogen. Specifically, in intensive care units (ICUs), *A. baumannii* is commonly isolated from the hospital environment and hospitalized patients. It causes nosocomial infections and has a high mortality rate (42–46). In addition to aminoglycosides and carbapenem, *A. baumannii* is resistant to some drugs.

Many enzymes have been identified in bacteria that cause antibiotic resistance. An example of such an enzyme is OXA-58, which hydrolyzes carbapenems. The Sec system translocates OXA-58 to the periplasm, where it is metabolized by OMVs. *A. baumannii* produces OXA-58 and OMV as CHDL (carbapenem-hydrolyzing class D-lactamases). OXA-58 in OMVs was confirmed by a carbapenem inactivation bioassay, proteomic analysis, and transmission electron microscopy following overexpression of enhanced green fluorescent protein fused to the OXA-58 signal peptide (18). Following Sec-dependent periplasmic translocation, most of the extracellular OXA-58 is released via OMVs; this suggests that *A. baumannii* OMVs contribute to antibiotic resistance spread and cause horizontal gene transfer of OXA-58 carbapenemase (47).

### Aggregatibacter actinomycetemcomitans

Among the virulence factors and mechanisms found in *Aggregatibacter actinomycetemcomitans* (*A. actinomycetemcomitans*) that colonizes the human oral cavity, this gram-negative, fastidious, non-motile bacterium is catalase-positive and oxidase negative. Localized aggressive periodontitis (LAP) is associated with this bacterium.

The virulence factor LtxA is a critical component of *A. actinomycetemcomitans*, as it causes the bacterium to colonize the host cell (48). In the Type 1 secretion system, LtxA is transmitted without requiring LFA-1 and cholesterol to reach the host cell (49). Furthermore, *Aactinomycetemcomitans* secretes LtxA using OMV. Due to the interaction between OMV and multiple host cell types, toxin activity increases when OMV transfers LtxA. A recent study found that OMVs from *A. actinomycetemcomitans* can transfer LtxA protein to target cells. The size of OMVs plays a direct role in the delivery of this protein (50).

As another virulence factor, the bacteria produce a cytolethal-distending toxin (CDT), which damages the host’s DNA. CDT targets the nucleus of a cell. The molecule comprises three subunits: CdtA, CdtB, and CdtC. Using liquid chromatography-tandem mass spectrometry, all three CDT subunits have been detected in OMV (22). *A. actinomycetemcomitans* OMVs have a mechanism for the delivery of biologically active CDT into susceptible cells. Future studies aim to treat localized aggressive periodontitis (LAP) by examining the means of transmission between OMV and LtxA and CDT and OMV (51).

### Commensal bacteria and *Bacteroides*

Various commensal bacteria inhabit the skin and mucous membranes of animals and provide vital services for their hosts. As a result, hosts receive calorie-rich energy, their immune systems grow, and they are protected against metabolic disorders (52). These bacteria have evolved to boost their multiplication through molecular communication. They can cause target cells to release OMVs with molecular payload (53).

The *Bacteroidetes* phylum is the most prominent gram-negative phylum in mammals’ gastrointestinal tracts (54). Human gut microbiota contains *Bacteroides* species, which are gram-negative and have well-established roles in communication, as their pathogenesis has been documented (55, 56).

Furthermore, they help maintain gut hemostasis, maintain host-commensal relationships, and are helpful for disease prevention (57). In addition, colitis can be prevented in people with inflammatory bowel disease (IBD) (58, 59). Several OMVs are produced by *Bacteroides fragilis* and *Bacteroides thetaiotaomicron* that have different protein compositions and are not made by bacterial lysis as by-products. Lipoproteins with hydrolyzing activity (glycosyl hydrolase) are selectively sorted into OMVs (60). A *Bacteroides* enzymatic lipoproteins assist in acquiring or breaking down polysaccharide complexes of fungi, plants, and mucins (61). *Bacteroides* can also produce many OMVs with different functional enzymatic activities, such as breaking down complex polysaccharides into simple forms and releasing them into the gut lumen so that other bacterial species can grow (microbiota) (62).

In contrast, some pathogens such as *Clostridium*, *Salmonella*, and *Campylobacter* can benefit from the glycosidic activity of OMVs. Bacteria with different metabolic abilities may also utilize these OMVs (63, 64). It is expected that these enzymes would change the nutrient content of the intestinal environment. However, these OMVs may increase producers’ colonization on the intestine’s surface. Bacteria sort proteins into OMVs by surface coupling and exposure. According to mass spectrometry data, Lipoprotein export sequences (LES) are critical in efficiently packing surface-exposed alpha-amylase SusG into Bacteroides OMVs (60). The proteins and LES of SusG may be excellent candidates for designing engineered *Bacteroides* strains that have medical properties in mammalian guts. Surface-exposed SusG in OMVs is active and can rescue the development of bacterial cells that cannot thrive on starch alone (65).

According to previous studies, *B. fragilis* benefits multiple sclerosis (MS) and IBD (66, 67). The bacterium produces capsular polysaccharide A (PSA), an immunomodulatory molecule that inhibits inflammation. As a result of the production of interleukin 10 (IL-10) and regulatory T cells, *B. fragilis* suppresses inflammation for the benefit of the host (68, 69). The PSA produced by these bacteria erodes intestinal immunity during normal colonization. According to some studies, OMVs are responsible for delivering PSA to the immune system, and this process occurs selectively because *B. fragilis* does not code secretion system genes (59, 70). Release of OMVs-included-PSA inhibits tumor necrosis factor (TNF-α) and T helper 17 (Th-17) development (59). Furthermore, they induce the differentiation of regulatory T cells by inducing dendritic cells to produce IL-10 (71). Toll-like receptor 2 (TLR2) detects OMVs-associated PSA in dendritic cells (DCs), resulting in the generation of anti-inflammatory cytokines and Tregs (72). In addition, OMV-induced signaling in DCs requires growth arrest and DNA-damage-inducible protein (Gadd45α), whereas Gadd45α-/-DCs cannot induce Treg responses and inhibit pro-inflammatory cytokines (59). It has been shown that DCs expressing Gadd45α are necessary for the activity of PSA-containing OMVs (59). Finally, produced- Treg can suppress the proliferation of T cells (73). OMVs containing PSA were shown to prevent experimental colitis, and *B. fragilis* was found to be a promising candidate for treating IBD (59).

### Bordetella pertussis

*Bordetella pertussis* is a gram-negative bacterium causing whooping cough. Among the significant factors contributing to *Bordetella pertussis* virulence are pertussis toxin, pertactin, adenylate cyclase toxin, dermo necrotic toxin, filamentous hemagglutinin (FHA), and BrkA. BvgAS is a two-component regulator system in *B. pertussis* that responds to extracellular stimuli. In *B. pertussis*, BvgAS consists of two components that react to extracellular stimuli. There is evidence that *B. pertussis* OMV can bind with lung A549 cells (74). A relationship between BVG+ OMV adhesions and the ability of anti-OMV serum to inhibit the adhesion of *B. pertussis* to lung epithelial cells has been demonstrated *in vitro* (75). As a result of their enrichment with Bvg+-activated adhesins, Bvg+ OMV exhibited the ability to adhere to human respiratory cells but not Bvg-OMV. A mouse aerosol challenge model has shown that immunization with recombinant BrkA significantly protected mice against lower respiratory tract infection with *B. pertussis*. Interestingly, only BrkA-expressing *E. coli* were inhibited by anti-OMV serum. BrkA and perhaps additional adhesins should be added to improve bacterial clearance following vaccination for the current acellular pertussis vaccines. *B. pertussis* OMVs provide antigens that are capable of explicitly targeting colonization. Compared with current aP vaccines, BrkA and possibly further adhesins might provide a higher level of protection, thereby improving the effectiveness of vaccination.

### Borrelia burgdorferi

The pathogenic bacterium *Borrelia burgdorferi* causes Lyme disease and is gram-negative and motile. The genome comprises 20 linear and circular plasmids and segmented linear chromosomes (76). Lyme disease is characterized by erythema migrans (EM), migratory inflammatory arthritis, and mono- or oligoarthritis, but EM lesions are the ones that confirm the diagnosis (77).

It has been reported that the OMV of *B. burgdorferi* contains two different porin activities (0.6 and 12.6 nano-Siemen). These activities are similar to those of porins in other spirochetes. OMV antigens, which include proteins on the bacterium’s surface, are associated with protective immunity and virulence. In addition to allowing the bacterium to attach initially, OMV also enhances EM development. The OMV of *B. burgdorferi* is composed of hydrophobic proteins, which are modified during *in vitro* passage. Consequently, these changes may be related to the ability of this bacterium to cause skin infection and EM by inducing an immune response. According to some studies, protein content in the OMVs of *B. burgdorferi* correlates with their pathogenicity (78).

### Burkholderia mallei

Glanders is a zoonotic infectious disease caused by *Burkholderia mallei*, a gram-negative bacterium. Glanders is a systemic disease that affects the nasal mucosa and the deep respiratory tract (79). In addition to being resistant to multiple antibiotics, this pathogen is a facultative intracellular bacillus.

OMV, including proteins, lipids, and carbohydrates, triggers an immune response. In a 2017 study, Sarah M. Baker found that the multilateral *B. pseudomallei* OMV vaccine provided cross-protection against inhalational glanders disease in mice and non-human primates (80). Inherently, OMV vaccines are safer than live attenuated vaccines because they are less complex. There is evidence that OMV induces IFN-producing T cells, and IFN- has been shown to suppress the growth of *B. mallei* infections in mice (80, 81). OMVs from *B. pseudomallei* can induce an immune response against glanders and myeloid cells by stimulating cellular and humoral immunity (81).

### Escherichia coli

*Escherichia coli*, a member of the Enterobacteriaceae family, is a facultative anaerobic Gram-negative bacterium that constitutes one of the most abundant species within the normal human gastrointestinal microbiota. While *E. coli* is typically recognized as a standard gut inhabitant, certain strains have developed pathogenic characteristics, enabling them to exhibit virulence (82–85). The adherent-invasive *E. coli* (AIEC) pathotype is hypothesized to play a role in the etiopathogenesis of (IBD, such as Crohn’s disease (CD) and ulcerative colitis (UC). AIEC strains are regarded as pathobionts, capable of inducing intestinal inflammation in predisposed individuals due to their genomic evolution and adaptation (86, 87).

OMVs derived from pathogenic *E. coli* strains play a crucial role in modulating host immune responses and disrupting intestinal barrier integrity, contributing to inflammatory diseases such as IBD (88, 89). Nadalian et al. (89) investigated the impact of OMVs from AIEC strain LF82 on intestinal epithelial cells (Caco-2). The study revealed that AIEC-derived OMVs upregulated Toll-like receptor (TLR-2, TLR-4) expression and altered the expression of junction-associated proteins (occludin, ZO-1, claudin-2, E-cadherin), potentially compromising epithelial barrier integrity. Additionally, OMV treatment induced a pro-inflammatory response, increasing the secretion of IL-8 and TNF-α, key cytokines in intestinal inflammation. These findings suggest that AIEC-derived OMVs contribute to epithelial dysfunction and inflammation, underscoring their potential role in the pathogenesis of IBD (89).

Hemorrhagic colitis and diarrhea can be caused by *Enterohemorrhagic Escherichia coli* (EHEC) O157. In addition, it can lead to hemolysis anemia, thrombocytopenia, and acute renal failure, which are all symptoms of a hemolytic uremic syndrome (HUS) (90). In the mice model, the OMVs of EHEC cause symptoms like HUS due to the presence of LPS and Shiga toxins (STx). OMVs appear to transmit LPS and STx from the gut lumen to the bloodstream in HUS patients (91). According to one study, eyedrop vaccinations with modified OMVs (mOMVs) from EHEC O157, containing a Penta-acylated lipid moiety that maintains subunits of non-toxic Shiga toxin B (STxB) but not STxA subunit proteins, can induce humoral and mucosal immune responses without any adjuvants and can serve as a protective vaccine against HUS symptoms (92).

Pro-inflammatory cytokines were induced by OMVs of the bacterium in intestinal epithelial cells. H7 flagellin is a component of EHEC OMVs that plays a significant role in inducing IL-8 production by activating the nuclear factor NF-κβ via Toll-like receptor 5 (TLR5). Additionally, it has been reported that the LPS present in these OMVs activates NF-κB signaling through TLR4 and myeloid differentiation Protein 2 (MD-2), which also leads to IL-8 production. Consequently, these OMVs may contribute to the pathogenic characteristics of this bacterium and could be utilized in the development of an effective vaccine, as they possess both pro-inflammatory and immunomodulatory properties (93).

Many causes of death are associated with septic shock, and septic-induced cardiomyopathy (SIC) is one of the most severe complications. It may be worsened by bacterial antibiotics due to the release of antibiotic-induced endotoxins. As inflammatory cytokines in the blood, OMVs can induce sepsis. Troponin T levels in the blood increased significantly during this process and resulted in the thickening of the heart wall and an increase in heart rate. There is evidence that OMVs can negatively affect the heart and cause injury to the heart. It has been reported that OMVs isolated from uropathogenic *E. coli* strains cause cardiac dysfunction even when bacteria are absent, so studies indicate that OMVs may be a potential therapeutic target for controlling sepsis (94).

The outbreak of diarrhea and HUS in 2011 was caused by *E. coli O104:H4*. There are several different virulence factors released by the OMVs of this bacterium. There are STx 2a, O104 LPS, H4 flagellins, and shigella enterotoxins in them. Apoptosis and the production of IL-8 are induced by OMVs attaching to intestinal epithelial cells through dynamin-dependent endocytosis and STx a2 independent endocytosis. Additionally, STX 2a plays a significant role in cytotoxicity, while flagellin and LPS are responsible for IL-8 production. A new method of delivering pathogenic factors to this bacterium via OMVs has been described in this report (95).

β-lactamase containing *E. coli* produces OMVs that include various subcellular antibiotic-resistant proteins. These OMVs degrade β-lactam antibiotics and support the survival of *E. coli* and other bacteria susceptible to these antibiotics. Specifically, OMVs contain antibiotic-resistant proteins and are necessary for bacteria to survive and grow in the presence of these antibiotics (96).

### *Francisella tularensis* and *Francisella novicida*

*Francisella tularensis* is a gram-negative, intracellular bacterium that invades macrophages. The highly virulent nature of *F. tularensis* makes it a helpful bioterrorism agent. In addition to serving as a key factor for macrophage survival and growth, the Francisella pathogenicity island (FPI) prevents the binding of phagosomes to lysosomes. Tularemia, a zoonotic disease, is caused by *Francisella tularensis* (97–99).

While *F. tularensis* is highly virulent, *Francisella novicida* is regarded as a less pathogenic species. However, it is frequently utilized as a model for investigating the infection mechanisms of *F. tularensis*. Proteins found in the OMVs of *F. novicida* have been associated with tularemia. Additionally, *F. novicida* can produce tubular extensions of its outer membrane in conjunction with its OMVs. *F. novicida* OMV and tubes (OMV/T) participate in pathogenesis and vaccine production, according to a recent study. Several factors contribute to OMV/T’s importance in pathogenesis: the vesicles contain antigens, secreted proteins, and virulence factors; the tubes are formed when *F. novicida* is inside the macrophage; OMV/T triggers the inflammatory response within the host cell. The production of OMV occurs during the exponential and early stationary phases of growth, but the production of OMV/T increases during the stationary phase (100).

Proteins and virulence factors can be secreted into the host cell via OMV/T. In the exponential phase, Fsp53 is the dominant secretory protein. It has been reported that deletion of the Fsp53 gene reduces macrophage infection and pathogenesis, but the function of the Fsp53 gene is unclear (101). Mutations in FtlA, a virulence gene secreted by OMV in *F. tularensis* LVS, cause a decline in infection in mice’s lungs. Lipase activity in FtlA allows it to bind to host cells effectively as an adhesion (102). It is unknown how OMV/T is produced, perhaps in a regulated manner. *Francisella* has OMV and tubes that regulate amino acid deprivation and central carbon metabolism. OMV/T production defects have been observed in hypo- and hyper-vesiculating mutants with mutations in fumA and tktA, which perform central carbon metabolism functions, as well as in FTN_0908 and FTN_1031, which perform unknown functions. In nutrient media, cysteine deficiency can increase the production of OMV/T *Francisella*. Thus, *Francisella* OMV plays an influential role in the cell. The OMV content can be an adhesive for pathogenic binding genes or attaching itself to the host cell. Amino acid deprivation and disorders of central carbon metabolism may play a role in OMV production, even though the mechanism is still unknown (97, 103, 104).

### Hemophilus influenzae

The upper and lower respiratory tract infections are caused by Non-Typeable *Hemophilus influenzae* (NTHi), a commensal, gram-negative bacterium found in the human nasopharynx. As a result of colonization, it causes respiratory tract infections, such as otitis media, sinusitis, and acquired pneumonia. It can also have significant consequences for chronic obstructive pulmonary disease (COPD) or cystic fibrosis patients.

In addition to providing immunity to these bacteria, NTHi OMVs are also responsible for their pathogenesis (105). Among the critical roles of NTHi OMVs are transforming DNA in bacterial cells (contributing to natural competence), evading human innate immune responses (106), increasing blood-brain barrier permeability, and maintaining biofilms (107). OMVs have a diameter of 20-200 nm and contain heme utilization protein, serine proteases, IgA endopeptidases, DNA, and adhesion P5 (106, 108–110). As a result of their attachment to human pharyngeal epithelial cells, NTHi OMVs aggregate on the surfaces of host cells in a time- and temperature-dependent manner, leading to their internalization (111). In these bacteria, OMVs are co-localized with the endocytosis protein caveolin, indicating internalization through caveolae (112). By interfacing with epithelial cells, OMVs release immunomodulatory cytokines, such as IL-8 and antimicrobial peptide LL-37, contributing to disease progression (1, 112).

### Legionella pneumophila

*Legionella pneumophila* is a gram-negative organism found in freshwater ecosystems and is an intracellular parasite of protozoa. It is responsible for legionnaire’s disease, severe form of pneumonia that can be fatal. Virulence factors are directly transferred to the extracellular environment by gram-negative bacteria via OMVs. *L. pneumophila* promotes the growth of Legionnaires’ disease in specific vacuoles after entering the human lung and infecting alveolar macrophages. Macrophages against *Legionella* infection, secretes TNF-α, IL-6 and IL-1β. Because *L. pneumophila* interferes with phagosome-lysosome fusion, it replicates in host organelles (113). By secreting OMVs, this phenomenon correlates with developmentally regulated changes in the LPS profile and inhibits phagosome-lysosome fusion. Phagosome-lysosome fusion is inhibited by *L. pneumophila* OMVs propagating into phagosomes (114, 115). OMVs from *L. pneumophila* can damage tissues and activate macrophages in a pro-inflammatory manner. Human macrophages were more susceptible to intracellular *L. pneumophila* replication following OMV pre-treatment, which increased the number of vacuoles per cell. Macrophages sense *L. pneumophila* OMVs via TLR2, which causes activation of macrophages acting via TLR2, IRAK-1, and NF-κB stimulate pro-inflammatory cytokines, while OMVs facilitate *L. pneumophila* replication by miR-146a-dependent IRAK-1 suppression. The miRNA-146a induced by OMVs facilitates bacterial replication (116).

Two sub secretary systems within *L. pneumophila* exist soluble supernatant proteins [SSPs] and OMVs. These are produced by proteins exiting the cytoplasm across the inner and outer membranes into the exterior space. The outer membrane of the bacterium is the source of OMVs. *L. pneumophila* virulence factors are found in the extracellular sub-secretory pathway (SSP) and OMV fractions. These components contain proteolytic and lipolytic enzymes that degrade human surfactant lipids, facilitating bacterial migration through the lung epithelium. In addition to degrading local matrices, OMVs further enhance bacterial migration. A notable virulence factor of *L. pneumophila*, associated explicitly with OMVs, is the macrophage infectivity potentiator (MIP), which may contribute to the destruction process. In the extracellular environment, it functions similarly to phospholipase C (PLC). Studies have demonstrated that serine metalloprotease, in conjunction with OMV-associated Mip, facilitates the penetration of Legionella pneumophila through epithelial and extracellular barriers in NCI-H292 lung epithelial cells (117).

### Moraxella

Infectious diseases caused by *Moraxella catarrhalis* (*M. catarrhalis*) include pneumonia in the elderly, septicemia, meningitis in adults, and acute otitis media in children. Currently, it is classified as a pathogenic bacterium since it is the third most common cause of acute otitis media in children. Moreover, it causes upper and lower respiratory tract infections in people suffering from chronic obstructive pulmonary disease (COPD), which can lead to pneumonia in the elderly and septicemia, meningitis, and endocarditis in the immunocompromised (118).

*M. catarrhalis* is one of the leading causes of inflammatory exacerbations in patients with COPD since it produces OMVs containing many pathogen-associated molecules (119, 120). There is a significant role for macrophages, epithelial cells, alveolar cells, and neutrophils in the pathogenesis of COPD diseases. A high level of pro-inflammatory cytokines and chemokines attracts neutrophils to the inflammation (121–124). OMVs of *M. catarrhalis* have been found to cause neutrophils to degranulate, causing tissue damage. During infection, OMVs of *M. catarrhalis* release LPS that deteriorates lung function, and they contain several virulence factors capable of evolving pro-inflammatory responses. Moreover, these OMVs induce pro-apoptotic cell death in A549 cells and stimulate IL-8 production by upregulating Intercellular Adhesion Molecule 1 (ICAM-1) expression, thereby contributing to inflammation and immune cell recruitment. Chang conjunctival cells, primary small airway epithelial cells (SAEC), type II pneumocytes (A549), and bronchial epithelial cells (BEAS-2B) can be infected with *M. catarrhalis*. As well as hiding in lymphoid tissue, this bacterium can reproduce in other tissues. *M. catarrhalis* OMVs cause cell death, hence breaches will occur in the epithelial barrier, and the bacterium will have the chance to pass through it, exacerbating COPD.

Multifunctional UspA1/A2 proteins are found in OMVs of *M. cataralis*, which interact with C3 (complement system third component). As a result, UspA1/A2 attaches to C4bBP and vitronectin and causes resistance to the bacteriolytic effects of the complement cascade in this bacterium. A comparison between OMVs with and without UspA1/A2 proteins has shown that OMVs with these proteins can neutralize the complement cascade by attaching to C3. Further, in a study of *M. catarrhalis* OMVs without UspA1/A2 proteins, there was a noticeable difference in the strength of the inhibitor of complement-dependent killing mechanisms of *H. influenzae*. Thus, the inactivation of C3 by *M. catarrhalis* can increase *H. influenzae* survival in the serum (3).

### Neisseria gonorrhoeae

*Neisseria gonorrhoeae* is a gram-negative diplococcus pathogen that causes sexually transmitted diseases such as gonorrhea. Gonorrhea is primarily caused by metabolites that impair macrophage and neutrophil function (125, 126). Previously, proteomics analysis of *N. gonorrhoeae* OMV revealed 110 proteins primarily derived from the outer membrane (127). Porin B (PorB), a key protein in *N. gonorrhoeae*, is critical in modifying the apoptosis process within mucosal membranes. *N. gonorrhoeae* is an aerobic, oxidase-positive bacterium capable of surviving inside neutrophils (128). PorB is expressed on the outer membrane of *N. gonorrhoeae*, and several proteins, including porins, are shared between the outer membranes of *N. gonorrhoeae* and its OMVs. Through its interaction with macrophages, PorB is transmitted to mitochondria via OMVs. N. gonorrhoeae utilizes OMVs as a secretion system to deliver virulence factors, contributing to its pathogenicity (127, 128). The PorB causes apoptosis in the genital mucosa by using OMV as a transmission pathway to the macrophage mitochondria (127).

The mechanisms governing the biogenesis of OMVs in *N. gonorrhoeae*, which exhibits a propensity for spontaneous OMV release at significantly higher rates than other gram-negative bacteria such as *Escherichia coli*, are not yet fully elucidated (127). The absence of Braun’s lipoprotein in *N. gonorrhoeae*, a protein that typically anchors the outer membrane to the peptidoglycan layer, is likely a contributing factor to the enhanced biogenesis of OMVs in this organism (128, 129). Furthermore, the downregulation of the phospholipid transporter MlaA, which occurs due to iron deficiency, leads to an enhancement in vesiculation within *Neisseria* species. Consequently, the biogenesis of OMVs is influenced by environmental conditions, particularly those present at mucosal surfaces (127, 130).

Dhital et al. (128) demonstrated that *N. gonorrhoeae* releases OMVs containing PorB porin and lipooligosaccharide (LOS), which interact with host immune cells, triggering inflammation and programmed cell death. Characterization of OMVs from four clinical isolates revealed conserved proteomic profiles but variations in OMV biogenesis and membrane component abundance. These differences influenced macrophage responses, with some isolates inducing higher IL-1α and IL-1β secretion, contributing to immune modulation and pathogenesis. This study highlights OMVs as key mediators of *N. gonorrhoeae* immune interactions, potentially affecting disease progression (128).

### Porphyromonas gingivalis

There are several gram-negative periodontal pathogens with high proteolytic activity, including *Porphyromonas gingivalis* (1). There is a strong link between periodontal disease and diabetes mellitus, atherosclerosis, heart disease, and autoimmune diseases such as rheumatoid arthritis (RA) in adults (3, 5). In some animal models, *P. gingivalis* OMVs induce inflammation characterized by the production of nitric oxide, the formation of macrophage foam cells, and the presence of neutrophils (1).

The OMVs of this bacterium contain several essential virulence factors, including gingipains, two types of LPS—O-LPS and A-LPS—fimbriae, GPG70, HBP35, and peptidyl arginine deiminase (PPAD) (1, 3). As far as topological species are concerned, there are three forms of PPAD: a soluble secreted form, a bound to OM form, and a bound to OMV form. PPAD associated with OMV and OM is altered by A-LPS. This OMV-bound form of PPAD is protected from proteolytic degradation, highlighting the crucial role of LPS modifications in the selective secretion and sorting of OMVs (5).

Epithelial cells are detached from the epithelium by OMVs containing antigens and proteases (gingipains-laden OMVs). It has been reported that the virulence factors of this bacterium are sorted selectively into OMVs. Furthermore, OMVs are internalized to the cells via lipid-raft defendant endocytic pathways and ultimately sorted into lysosomes. By lysis of OMVs, antigen-presenting cells (APCs) like dendritic cells and macrophages can recognize these released antigens, inducing adaptive immunity (pathogen-specific antibodies). In the animal model (mice), it has been demonstrated that OMVs in *P. gingivalis* are instrumental in enhancing the antigenicity of this bacterium, and intranasally administered OMVs effectively induce serum IgG and IgA and salivary IgA (4).

### Pseudomonas aeruginosa

As an opportunistic gram-negative pathogen, *P. aeruginosa* can cause serious infections in immunocompromised patients and those with cystic fibrosis. The bacterium is intrinsically and adaptively resistant to antibiotics (3, 131, 132). *H. pylori* and *P. aeruginosa* affect the interactions between host and pathogen. Both bacteria activate both the canonical and non-canonical inflammasome pathways. Despite this, OMVs failed to activate inflammasomes because this bacterium has a modified type of LPS that has only weak biological activity but can be weakly recognized by caspase11 (1).

According to studies, OMVs of *P. aeruginosa* induce inflammasomes in mouse macrophages, resulting in the formation of specks and cleavage and secretion of caspase-1 and interleukin-1β (133, 134). These responses were reported to be dependent on non-canonical caspase 11, while they were independent of interferon-inducible protein (AIM2) and NOD-like receptor family CARD domain-containing protein 4 (NKRC4) canonical inflammasomes. Inflammasome activation can also be achieved without exogenous priming signals by TLR-dependent mechanisms. As demonstrated in human monocytes, caspase 5 is necessary to activate the inflammasome. It is interesting to note that caspase 4 was needed to activate the inflammasome after cells were exposed to free LPS from *P. aeruginosa*. As a result, caspase 4 and caspase 5 recognize LPS differently based on LPS’s physical form or delivery. Results from this study demonstrate a correlation between OMVs, host-pathogen interaction, and infections in humans, as well as the possibility of using these OMVs as novel vaccines (1, 133, 134).

Numerous studies have demonstrated that OMVs derived from *P. aeruginosa* interact with cholesterol-enriched lipid rafts in the apical membrane, facilitating the transfer of specific virulence factors, such as CIF, into the cytoplasm. This process results in a decreased production of wild-type cystic fibrosis transmembrane conductance regulator (wt-CFR) chloride ions (135, 136). In addition, cyclodextrin is FDA-approved for solubilizing lipophilic drugs (137) and disorganizes lipid rafts by reducing the cholesterol part of the cell membrane. Besides blocking the internalization of *P. aeruginosa* into epithelial cells of the lungs, it also blocks the fusion of OMVs *P. aeruginosa* with A549 cells. Studies have reported that cyclodextrins inhibit *P. aeruginosa* OMVs from secreting CFR Cl- or forming biofilms and that this can be useful for patients with cystic fibrosis (4, 138).

Ge et al. (139) investigated the effects of *P. aeruginosa* OMVs on lung epithelial cells, revealing that Pseudomonas aeruginosa OMVs induce oxidative stress and autophagy while also activating the NLRP3 inflammasome, a key regulator of inflammation. Inhibition of NLRP3 using MCC950 further enhanced *P. aeruginosa* OMV-induced autophagy, suggesting that NLRP3 suppresses autophagy to some extent. Additionally, *P. aeruginosa* OMVs increased AMP-activated protein kinase (AMPK) expression, which regulates cellular energy homeostasis. These findings indicate that *P. aeruginosa* OMVs modulate autophagy and inflammatory responses, providing insights into their potential therapeutic applications (139).

### Yersinia pestis

In addition to delivering some virulence factors of pathogenic bacteria during infection, OMVs are released from gram-negative bacteria during the division and growth of cells (1). Insects and mammals can get sick from *Yersinia pestis*, a gram-negative bacterium. Inhalation of aerosols or respiratory droplets and bites from hematophagous insects can transmit this bacterium to its hosts. Among the diseases caused by this pathogenic bacterium are the septicemic plague, bubonic plague, and pneumonia (3, 140, 141). *Yersinia pestis* possesses a variety of virulence factors, including plasminogen activator, which plays a crucial role in the pathogenesis of both bubonic and pneumonic plague. Pla facilitates the activation of plasminogen and degrades alpha-2-antiplasmin (α2AP) as well as the Fas ligand (FasL) by cleaving host proteins, thereby contributing to immune evasion and tissue invasion. Additionally, this protein can act as an adhesion to attach this bacterium to the extracellular matrix and cause the bacterium to invade HeLa cells (142, 143). In physiological conditions, *Yersinia pestis* produces OMVs containing proteins associated with virulence factors such as F1 outer fimbrial antigen and adhesion Ail. A direct relationship exists between temperature and the amount of OMVs-associated proteins. A further difference is that these proteins increase at 37°C rather than at 25°C. In addition to mutations in RseA, Hfq, and the essential Braun lipoprotein (Lpp), membrane stress can also enhance the expression of these proteins. Furthermore, extracellular matrix components such as laminin and fibronectin have been reported to bind to the OMVs of *Yersinia pestis*. OMVs containing the Pla protein are also implicated in modulating the infection outcome by interacting with FasL and plasminogen (1, 144).

### Helicobacter pylori

*Helicobacter pylori* is a gram-negative, microaerophilic, spiral bacterium that colonizes the human gastrointestinal tract and causes acute and chronic gastritis, gastroduodenal ulcers, and gastric malignancy. *H. pylori* infects at least half of the world’s population. Clinical symptoms are significantly influenced by *H. pylori’s* interaction with host cells (145–149). *H. pylori*, an extracellular bacterium characterized by limited invasiveness, predominantly inhabits the gastric mucus layer, with only a minor fraction adhering to the epithelial cell surface. Current evidence does not support the notion that *H. pylori* can penetrate the bloodstream. Consequently, virulence factors derived from *H. pylori*, particularly OMVs, may significantly contribute to the pathogenesis of the documented extra-gastric diseases. Recent studies suggest that OMVs originating from the host microbiota, especially those produced by commensal bacteria within the gastrointestinal tract, can enter the circulatory system (150–153). *H. pylori* consistently releases OMVs *in vivo* and *in vitro* (154, 155). A recent investigation revealed that the transcriptomic alterations induced by *H. pylori* OMVs in the MKN74 gastric adenocarcinoma cell line closely resemble those elicited by the parent bacterium. This suggests that OMVs may significantly contribute to or enhance the pathogenic effects of *H. pylori* (156). Furthermore, the identification of *H. pylori*-derived OMVs in the serum of mice infected with *H. pylori* offers additional support for the potential role of these OMVs in extra-gastric diseases (157).

In *H. pylori*-OMV, 162 OMV-associated proteins have been identified in strain J99 and 91 in strain 11637 through proteomic analysis. There are many molecules in OMV, including CagA, VacA, OMP porins, HpaA, OMP18, NapA, peptidyl-prolylcis-trans-isomerase, gamma glutamyltranspeptidase, OipA, and Hsp60, capable of eliciting immunological responses (153, 158).

Biofilm formation on the human gastric mucosa is a critical pathogenic marker of *H. pylori*. The extracellular matrix of these biofilms comprises a complex mixture of exopolysaccharides, proteins, DNA, and other macromolecules. The *H. pylori* strain TK1402 exhibits a strong biofilm-forming ability, as demonstrated by SDS-PAGE analysis (159). It has been hypothesized that OMV-associated proteins are essential for biofilm formation in *H. pylori*. Among the key adhesion factors, AlpA and AlpB facilitate laminin-binding and contribute to gastric injury by mediating bacterial adhesion to the gastric epithelium. Notably, AlpB plays a crucial role in biofilm formation, and sequence variations in the *alpB* gene influence both biofilm formation and bacterial adhesion to gastric cells. *H. pylori* utilizes OMVs to directly transfer virulence factors to the host, further enhancing its pathogenic potential. Additionally, biofilm formation in the gastric mucosa is associated with OMV-mediated bacterial colonization, which may play a role in persistent infection and bacterial survival strategies (160). Yonezawa et al. (161) established that the formation of biofilms by *H. pylori* is contingent upon cell-cell aggregation facilitated by OMVs. Furthermore, OMVs were identified within the extracellular polymeric substance matrix of the TK1402 clinical isolate, which exhibits a pronounced capacity for biofilm formation. The introduction of OMVs derived from TK1402 into *H. pylori* cultures significantly augmented biofilm development (161).

In addition to protein molecules, extracellular DNA (eDNA), primarily associated with the surface of OMVs, appears to play a crucial role in developing biofilms formed by *H. pylori*. This bacterium produces OMVs in biofilm (bOMVs) and planktonic (pOMVs) states. In the *H. pylori* strain NCTC11639, bOMVs exhibit a broader size distribution, a more significant negative charge, enhanced aggregation of OMVs, and a fourfold increase in eDNA content compared to pOMVs. These findings suggest that bOMVs may serve to protect eDNA from degradation. Consequently, eDNA may function as a bridging agent, facilitating OMV-OMV and cell-cell aggregation, thereby promoting the formation of biofilms (162). Recent findings indicate that the α-class carbonic anhydrase (α-CA), a periplasmic enzyme crucial for the acid acclimatization of *H. pylori* in the human stomach, has been identified in bOMVs and pOMVs from four distinct strains of *H. pylori*. Notably, the concentration of α-CA was found to be significantly higher in pOMVs compared to bOMVs. Furthermore, it has been established that α-CA facilitates the release of eDNA, which plays a role in enhancing biofilm stability (163).

Previous research has consistently demonstrated that OMVs derived from *H. pylori* promote the production of IL-8 by gastric epithelial cells in a dose-dependent manner, both *in vivo* and *in vitro* (155, 164, 165). IL-8 functions as a pro-inflammatory cytokine and chemokine, facilitating the recruitment of immune cells, including macrophages, neutrophils, and T lymphocytes, to the affected tissues, subsequently leading to a pronounced mucosal inflammatory response (166). Kaparakis et al. (167) provided evidence that the delivery of peptidoglycan via OMVs is responsible for activating cytosolic NOD1-dependent NF-κB signaling and subsequent IL-8 production in AGS cells, as opposed to LPS. Notably, the microinjection of peptidoglycan directly into AGS cells did not elicit a NOD1-dependent inflammatory response, thereby underscoring the significant role of *H. pylori* OMVs in facilitating the delivery of bacterial peptidoglycan to provoke inflammation in host cells (167). Notably, a separate study has shown that OMVs play a role in the transport of LPS into the cytosol of host cells, which subsequently activates caspase-11-dependent pyroptotic cell death and IL-1 responses (5). These findings highlight the crucial role of H. pylori OMVs in bacterial pathogenesis, contributing to biofilm formation, immune modulation, and inflammation.

### Campylobacter jejuni

*Campylobacter jejuni* is a gram-negative, microaerophilic, nonfermenting, and oxidase-positive bacterium. It is a major human pathogen responsible for causing dysentery and gastroenteritis, as well as contributing to intestinal inflammation, Guillain-Barré syndrome, and arthritis. The infection process begins with the colonization of the intestinal mucosa, followed by the invasion of human intestinal epithelial cells (IECs*)* (168, 169).

The cytolethal distending toxin (CDT) is one of the most essential factors in the pathogenesis of *C. jejuni* transmitted by OMVs. The CDT consists of three subunits: CdtA, CdtB, and CdtC. The CdtA and CdtC subunits deliver CdtB into host cells as carriers. DNA repair responses are activated, and cell cycle arrest occurs at the G2/M phase with the action of CdtB as a DNase. CDT response from *C. jejuni* results in the production of IL-8, IL-6, and hBD-3 from intestinal epithelial cells (IECs) of type 84. In previous studies, OMVs from *C. jejuni* induced IL-8 through a mechanism that is not dependent on CDT. The role of N-linked glycosylation in *C. jejuni* is still under investigation, but it may be involved in evading host immune response based on a recent investigation of a large number of N-linked glycoproteins associated with *C. jejuni* OMVs (170–172).

Based on Elmi et al. research, *C. jejuni* OMVs have a proteolytic activity which contains three proteases, HtrA, Cj0511, and Cj1365c. The role of proteases in homeostasis cannot be overstated. Bacteria interact with host cells and cleave E-cadherin or occludin by HtrA and Cj1365c proteases. In bacterial colonization, Cj0511 proteases play an influential role. *C. jejuni* OMVs possess proteolytic activity that enhances bacterial invasion of intestinal epithelial cells via E-cadherin and occludin (173).

As reported by Abdi Elmi et al., bile salt sodium taurocholate (ST) stimulates the production of Jejuni OMVs and boosts their proteolytic activity. As a signal, bile, one of the host metabolites, modulates the global gene expression of virulence factors. It appears that ST is implicated in the pathogenesis of *C. jejuni* due to its activation of the protein content of OMVs, such as up-regulation of HtrA, Cj0511, Cj1365c, and CDT genes. The ST actually enhances the cytotoxicity and immunogenicity of OMVs against IECs. Furthermore, ST, SD (bile salt sodium deoxycholate) induces *Campylobacter* invasion antigen (Cia) genes, resulting in an enhanced invasion of IECs by *C. jejuni*. Infection of IECs by *C. jejuni* may be enabled by Cia proteins secreted by OMVs (174).

### Vibrio cholerae

*Vibrio cholerae*, the causative agent of cholera, is a Gram-negative bacillus primarily transmitted through contaminated water. Each year, *V. cholerae* is responsible for approximately 120,000 deaths worldwide. Based on serotypic classification, *V. cholerae* is divided into two major groups: O1 and non-O1. The O1 serogroup is further subdivided into two biotypes: Classical and El Tor (175–177). The primary virulence factors of *V. cholerae* include cholera toxin and the toxin-coregulated pilus (TCP). Cholera toxin induces severe watery diarrhea by increasing intracellular cyclic AMP (cAMP) levels. In addition to these significant virulence factors, *V. cholerae* produces hemagglutinin protease and serine protease (VesC), both of which contribute significantly to its pathogenicity (176).

Recent studies have shown the presence of HAP and VesC in the *V. cholera* OMVs. OMVs secrete biologically active proteases, which may play a role in cytotoxic and inflammatory responses (178). Several *Vibrio cholerae* virulence factors, such as CT, *Vibrio cholera* cytolysin (VCC), and PrtV, are actively secreted by OMV (179). The VesC stimulates T84 cells and produces IL-8, so it plays an essential role in the inflammatory response (180).

OMV in *Vibrio cholerae* is made spontaneously from LPS and outer membrane proteins, activating the secretion of proteases (181). OMVs in *Vibrio cholerae* function as heat-resistant immunogens crucial in eliciting an immune response. These OMVs stimulate the production of toll-like receptor 4 (TLR4) and toll-like receptor 2 (TLR2), leading to acute inflammation and a toxic immune response in the host (182).

The toxicity of *Vibrio cholerae* OMVs is a significant concern in vaccine production. Cholera pentavalent outer membrane vesicles (CPMVs (have recently emerged as suitable candidates for the *Vibrio cholerae* vaccine. According to research, All-trans Retinoic Acid (ATRA) neutralizes the toxic effects of OMV on CPMVs without modifying OMV antigenicity (183). ATRA activates vitamin A metabolite, which can reduce the expression of inflammatory factors IL-6, IL-12, and TNF by modulating NFjb signaling and also down-regulating TLR2-mediated pro-inflammatory cytokine response. Mucosal vaccines have a significant effect on mucosal infections. High doses of CPMVs are more effective than low doses. As a potent adjuvant with CPMVs in oral vaccines, ATRA induces proper behavior of intestinal lymphocytes (183, 184).

## OMVs and cancer

Worldwide, cancer continues to be a serious problem. Even though cancer is not an infectious illness, 13% of cancers worldwide are believed to be caused by infectious microorganisms such as bacteria, viruses, and fungi (13, 146, 185, 186). The formation of several malignant tumors, including esophageal, breast, gastric, colorectal, and oral cancers, is substantially influenced by the dysregulation of bacterial-host interactions (13, 187, 188). Since OMVs are recognized as an essential communication mediator between bacteria and host cells, their information transmission and regulatory roles in carcinogenesis and development have garnered more attention in recent years (189). It has been shown by OMV kinetic studies that OMVs that enter the body may move through the host’s circulatory system to various organs, participating in the long-distance signal transmission between bacteria and host organs *in vivo* (190–192).

Further investigations have demonstrated that OMVs can accumulate within the tumor microenvironment (TME). In their study, Kim et al. (193) utilized constructs of *E. coli*-derived OMVs that were fluorescently labeled with Cy7 for injection into tumor-bearing murine models. The findings from *in vivo* fluorescence imaging indicated that OMVs exhibited the more significant accumulation in tumor tissues, including those associated with colon adenocarcinoma, melanomas, and breast cancer, compared to other organs (193). Kuerban et al. (194) identified a comparable occurrence in a murine model of non-small cell lung cancer (NSCLC), wherein OMVs derived from *Klebsiella pneumoniae* exhibited a greater fluorescence intensity in tumor tissue compared to other organs, indicating a preferential uptake by the neoplastic tissue (194).

The instances above represent a non-exhaustive overview of the accumulation of OMVs within tumors. Contemporary perspectives indicate that the enhanced permeability and retention (EPR) effect plays a significant role in the localization of vesicles at tumor sites. This phenomenon is attributed to the improved vascularization of tumor tissues, the reduced presence of lymphatic vessels, and the more loosely organized arrangement of endothelial cells within the blood capillaries. These characteristics facilitate the efficient infiltration of specific liposomes and nanoparticles into the tumor vasculature, enabling them to persist within the tumor site while evading removal through lymphatic drainage (195, 196).

OMVs facilitate the transmission of virulence factors, allowing them to affect the genetic stability and function of host cells. VacA can reduce its transmission from bacteria to cells when carried by OMVs, as Chitcholtan et al. (197) showed, this can result in micronuclei formation and increase *H. pylori’s* carcinogenic potential (197). In a recent study, Turkina et al. utilized proteomic and imaging techniques to demonstrate that OMVs from *H. pylori*, which contain the CagA protein, can facilitate ATP binding to histone H1 in epithelial cells. This interaction subsequently leads to modifications in chromosomal remodeling and may initiate tumorigenesis (198).

OMVs derived from *E. coli* have been shown to contribute to carcinogenesis and enhance the ability of bacteria to penetrate epithelial cells (199, 200). Research conducted by Tyrer et al. (201) indicated that OMVs can be internalized by Caco-2 cell lines, leading to the induction of DNA double-strand breaks and aneuploid replication in these cells, as evidenced by the tracking of fluorescent markers associated with *E. coli*-produced OMVs. Their findings highlight the genotoxic effects of *E. coli*-derived OMVs on host cells, thereby suggesting a potential link to carcinogenic processes (201).

Also, OMVs derived from *E. coli* have shown potential in cancer therapy due to their ability to modulate tumor cell behavior and induce apoptosis, making them a promising strategy for colorectal cancer treatment. Jiang et al. (2024) investigated the therapeutic effects of *E. coli* OMVs on CT26 colon carcinoma cells. The study demonstrated that OMVs inhibited tumor growth in mice and significantly increased apoptosis in CT26 cells by upregulating Bax expression and downregulating Bcl-2, thereby reducing the Bcl-2/Bax ratio. *In vitro*, E. coli OMVs entered CT26 cells, reducing proliferation, migration, and invasion. These findings suggest that *E. coli* OMVs exert anti-tumor effects by inducing apoptosis, highlighting their potential as a novel therapeutic approach for colorectal cancer (202).

## Discussion

OMVs are closed, spheroid particles that range in size from 10 to 300 nm in diameter and are naturally released by Gram-negative bacteria during all phases of growth. To date, all examined Gram-negative bacteria have been shown to produce OMVs. These vesicles contain a diverse array of bacterial components and can significantly influence both the host organism and the immune system. Their effects may contribute to disease progression, inflammation, and immune evasion (18, 41, 203). Given these characteristics, OMVs hold potential applications in various fields, including:

### OMVs as vaccines

Nowadays, vaccine expansion is the most active research field in biomedical sciences (204–206). The persistent development of vaccines is essential to prevent the advent of new infectious, OMVs have been involved in lots of variety carrier functions, although, OMVs have great potential as innate vaccines (204). *In vivo*, OMVs have a wide range of interactions with immune cells that show their potential to be used for immunization (207, 208). The first studies demonstrated immune responses elicited by OMVs promising inductions of cytokines and chemokines in macrophages and other cell types. A study showed that small outer membrane vesicles (sOMVs) derived from *E. coli* induce the expression of CXCL1 in mouse endothelial cells, leading to increased infiltration of neutrophils (209). This finding was further confirmed for several other heterologous antigens loaded into *E. coli* genetically engineered OMVs (gOMVs), highlighting their ability to preserve the native structure of antigens within OMVs (210). Several studies have investigated the immunization potential of OMVs in mice, demonstrating their protective effects against subsequent infections. For example, immunization with *Vibrio cholerae* sOMVs in mice induced immunoglobulin production and conferred protection against this bacterium in their offspring (211). Similarly, studies on *E. coli* sOMVs in mice revealed that immunization protected sepsis primarily by inducing T-cell-mediated immunity (212). Additionally, in the case of *Shigella flexneri*, immunization with merged sOMVs successfully protected mice from a subsequent lethal *Shigella* challenge (213). Moreover, two kinds of OMV-based vaccines against *Neisseria meningitidis* are currently the only OMV-based vaccines licensed (214).

### OMVs as a reaction scaffold

In one study, E. coli OMVs were utilized as a scaffolding platform for facilitating cascading cellulose hydrolysis reactions. Many anaerobic bacteria transport cohesin-dockerin complexes on their cell surface to mediate cellulose hydrolysis. In this study, E. coli OMVs were genetically engineered to display three distinct cohesin domains derived from different organisms, along with a membrane anchor and a cellulose-binding module. The interaction of these cohesin domains with their specific docker in molecules permitted the assembly of three cellulases on the vesicle surface and increased sugar hydrolysis by 29-fold compared with dissolve enzymes. Cellulose hydrolysis was chosen as a proof of principle, but OMVs could in point be utilized as scaffolding platforms for any desired enzymatic reaction cascade to modify reaction rate and product efficiency, making OMVs natural nanoreactors (7).

### Vesicles as specialized drug delivery vehicles

A promising new approach used OMVs with low immunogenicity and carried an antibody targeting them to cancer cells; these OMVs also carried small interfering RNA, which led to gene silencing and consequently caused tumor regression in a mouse model (7). If such usages of engineered OMVs for cancer therapy carry on to be successful in animal patterns, they have the potential to be helpful in the growth of targeted human therapeutics.

## RNA components in OMVs and their functional roles

Recent research has shown that OMVs encapsulate mRNA and sRNA molecules in addition to proteins, lipids, and DNA. OMVs generated from different gram-negative bacteria have been shown to include these mRNAs and sRNA components, indicating that they could constitute a crucial intercellular communication mechanism (32, 215). Recent research has shown that bacterial vesicles containing noncoding regulatory RNAs are released into the environment and spread to other microorganisms and host cells, as has already been documented for the protozoan pathogen *Trypanosoma cruzi*. This highlights the importance of microbial sRNAs as molecules that can mediate host-microbe interactions. Nevertheless, sRNAs with regulatory roles comparable to those of miRNAs can also be produced by intracellular bacterial pathogens (216, 217). Since extracellular sRNAs are present in many body fluids, including serum, plasma, and urine, and since their levels in the blood are changed in several illnesses, they may be used as biomarkers for pathological conditions (218).

Many of these sRNAs are categorized into distinct functional groups by their designated biological functions (219). Numerous regulatory mechanisms are present in bacterial sRNAs. Bacterial sRNAs can attach to protein targets and alter their activities. For example, the RNAs *CsrB* and *CrsC* bind to the CsrA protein and decrease its activity by separating it from its targets, or the RNA *MicF* of *E. coli* suppresses the development of the outer membrane protein *OmpF* (220, 221). Bacterial sRNAs can attach to the Hfq protein, which is comparable to the RISC complex in eukaryotes, and use RNA base pairing to control the production of target mRNAs. A very abundant and conserved protein, Hfq has a role in several RNA-mediated processes. Lastly, the ribosome binding site can be blocked or unmasked by sRNAs (222–224).

As previously documented by the protozoan pathogen *Trypanosoma cruzi*, bacterial sRNAs (i.e., tRNA fragments) may be internalized into extracellular vesicles, released in the surrounding environment, and transmitted to other microorganisms and host cells (225–227). Nevertheless, sRNAs with regulatory roles comparable to those of miRNAs can be produced by intracellular bacterial pathogens. Indeed, following *Mycobacterium marinum* infection of human THP-1 macrophage cells, the scientists noticed that sRNAs capable of binding the host RNA-induced silencing complex (RISC) were produced, interfering with miRNA-mediated post-transcriptional gene regulation (228). Additionally, pathogenic sRNAs from the fungal disease Botrytis cinereal can bind RISC and suppress host-immunity genes (229). Furthermore, it has been shown that the sRNA *PinT* generated by the intracellular pathogen *Salmonella enterica* controls host gene expression and mediates the activity of virulence genes and invasion-associated bacterial effectors necessary for intracellular survival (230).

Lastly, miRNA-sized sRNAs (msRNAs) produced by periodontal pathogens have been shown to be able to be packed in OMVs, transported into eukaryotic cells (such as T lymphocytes), and trigger the production of cytokines including IL-5, IL-13, and IL-15 (231). Likewise, OMVs can transfer methionine tRNA, which is generated from Pseudomonas aeruginosa, into human epithelial airway cells, hence reducing the release of IL-8. Consequently, each of these investigations highlights the significance of microbial sRNAs as essential communication molecules capable of mediating interactions between microbes and their hosts (31, 216).

**Table 1** provides a comprehensive list of OMV-associated proteins, toxins, and their targets across various bacterial species, offering a clearer understanding of the role of OMVs in bacterial pathogenesis. **Table 2** summarizes the modes of action induced by the OMVs of different bacterial species, including pro-inflammatory, anti-inflammatory, and apoptotic effects, and the key virulence factors associated with each species.

**Table 1:** Summary of OMV-associated proteins, toxins, and targets in different bacterial species.

Bacterial Species	OMV Proteins	Toxins	Targets/Functions	OMV production method	Reference
*Escherichia coli*	H7 flagellin, LPS	Shiga toxins (Stx)	Inducing IL-8 production, immune activation	OMVs are produced during the exponential and stationary phases of growth. Heat treatment followed by ultrafiltration.	(92, 93, 232)
*Helicobacter pylori*	CagA, VacA, OipA	VacA, Hsp60	Inflammation, gastric ulcer formation	OMVs are secreted during the late logarithmic and early stationary phases.	(153, 158, 233)
*Neisseria gonorrhoeae*	PorB, Opa	LOS (lipooligosaccharides)	Host cell apoptosis, immune evasion	OMVs are produced upon bacterial activation in response to host-cell interaction.	(127, 128)
*Pseudomonas aeruginosa*	AlgU, OprF, OprD	Exotoxin A, elastase	Biofilm formation, immune response modulation	OMVs are produced during the stationary phase and are influenced by quorum sensing.	(234, 235)
*Vibrio cholera*	OmpU, VesC	Cholera toxin (CT), VCC	Toxin delivery, inflammatory response	OMV production increases under nutrient-deprived conditions.	(236–238)
*Francisella tularensis*	FtlA, Fsp53, fumA	FPI-related factors	Macrophage invasion, immune evasion	OMVs are produced during the exponential growth phase and increase under nutrient or amino acid deprivation, with defects observed in mutants of fumA and tktA.	(101, 102, 239)
*Campylobacter jejuni*	HtrA, Cj0511, Cj1365c	Cytolethal distending toxin (CDT)	IEC invasion, immune evasion	OMVs are produced during the late exponential and stationary phases.	(173, 240–242)

**Table 2:** Mode of action induced by OMVs in different bacterial species.

Bacterial Species	Pro-inflammatory/Anti-inflammatory/Apoptotic Effects	Virulence Factors	Reference
*Escherichia coli*	Pro-inflammatory (IL-8 production via NF-κB activation)	Shiga toxins (Stx), Flagellin, LPS	(92, 243)
*Helicobacter pylori*	Pro-inflammatory (Gastric ulcer formation, immune modulation)	CagA, VacA, OipA	(153, 158, 233)
*Neisseria gonorrhoeae*	Apoptotic (Induces apoptosis in host cells via PorB)	LOS	(127, 128)
*Pseudomonas aeruginosa*	Pro-inflammatory (Biofilm formation, immune response modulation)	Exotoxin A, elastase	(234, 235)
*Vibrio cholera*	Pro-inflammatory (IL-8 production, inflammatory response)	Cholera toxin, VesC	(236, 237)
*Francisella tularensis*	Apoptotic (Macrophage invasion, immune evasion)	FtlA, Fsp53, FPI-related factors	(101, 102)
*Campylobacter jejuni*	Pro-inflammatory (IL-8 production)	Cytolethal distending toxin (CDT)	(173, 240)

## Conclusion

OMVs are critical mediators of bacterial pathogenesis, influencing infection outcomes through immune evasion, biofilm formation, and horizontal gene transfer. Their ability to transport virulence factors, toxins, and regulatory molecules highlights their role in host-pathogen interactions. While OMVs are well-recognized contributors to bacterial virulence, they also present promising avenues for biomedical applications, including vaccine development, targeted drug delivery, and immunotherapy.

Despite significant advancements in OMV research, several questions remain unanswered. Future studies should focus on elucidating the precise mechanisms of OMV biogenesis and cargo selection, which could aid in designing OMV-based therapeutic strategies. Additionally, investigating the impact of OMVs in polymicrobial infections and their role in antibiotic resistance could provide new insights into bacterial adaptation and survival. The potential of engineered OMVs in vaccine development warrants further exploration, particularly in the context of infectious diseases and cancer immunotherapy. Understanding these aspects will contribute to developing novel interventions for combating bacterial infections and improving human health.
